# Network analysis of the relationship between depressive symptoms, demographics, nutrition, quality of life and medical condition factors in the Osteoarthritis Initiative database cohort of elderly North-American adults with or at risk for osteoarthritis

**DOI:** 10.1017/S204579601800077X

**Published:** 2019-02-06

**Authors:** Marco Solmi, Ai Koyanagi, Trevor Thompson, Michele Fornaro, Christoph U Correll, Nicola Veronese

**Affiliations:** 1 Neuroscience Department, Psychiatry Unit, University of Padua, Padua, Italy; 2 Padua University Hospital, Padua, Italy; 3 Neuroscience Center, University of Padua, Padua, Italy; 4 Instituto de Salud Carlos III, Centro de Investigación Biomédica en Red de Salud Mental, CIBERSAM, Monforte de Lemos 3-5 Pabellón 11, Madrid 28029, Spain; 5 Research and Development Unit, Parc Sanitari Sant Joan de Déu, Universitat de Barcelona, Fundació Sant Joan de Déu, Dr Antoni Pujadas, 42, Sant Boi de Llobregat, Barcelona 0883, Spain; 6 Faculty of Education and Health, University of Greenwich, London, UK; 7 Neuroscience Department, Section of Psychiatry, Federico II University of Naples, Naples, Italy; 8 Department of Psychiatry, The Zucker Hillside Hospital, Northwell Health, Glen Oaks, NY, USA; 9 Department of Psychiatry and Molecular Medicine, Hofstra Northwell School of Medicine, Hempstead, NY, USA; 10 Department of Child and Adolescent Psychiatry, Charité Universitätsmedizin, Berlin, Germany; 11 Ageing Branch, National Research Council, Padua, Italy

**Keywords:** Depressive symptoms, elderly, functioning, income, network analysis, quality of life

## Abstract

**Aims:**

A complex interaction exists between age, body mass index, medical conditions, polypharmacotherapy, smoking, alcohol use, education, nutrition, depressive symptoms, functioning and quality of life (QoL). We aimed to examine the inter-relationships among these variables, test whether depressive symptomology plays a central role in a large sample of adults, and determine the degree of association with life-style and health variables.

**Methods:**

Regularised network analysis was applied to 3532 North-American adults aged ⩾45 years drawn from the Osteoarthritis Initiative. Network stability (autocorrelation after case-dropping), centrality of nodes (strength, M, the sum of weight of the connections for each node), and edges/regularised partial correlations connecting the nodes were assessed.

**Results:**

Physical and mental health-related QoL (*M* = 1.681; *M* = 1.342), income (*M* = 1.891), age (*M* = 1.416), depressive symptoms (*M* = 1.214) and education (*M* = 1.173) were central nodes. Depressive symptoms’ stronger negative connections were found with mental health-related QoL (−0.702), income (−0.090), education (−0.068) and physical health-related QoL (−0.354). This latter was a ‘bridge node’ that connected depressive symptoms with Charlson comorbidity index, and number of medications. Physical activity and Mediterranean diet adherence were associated with income and physical health-related QoL. This latter was a ‘bridge node’ between the former two and depressive symptoms. The network was stable (stability coefficient = 0.75, i.e. highest possible value) for all centrality measures.

**Conclusions:**

A stable network exists between life-style behaviors and social, environmental, medical and psychiatric variables. QoL, income, age and depressive symptoms were central in the multidimensional network. Physical health-related QoL seems to be a ‘bridge node’ connecting depressive symptoms with several life-style and health variables. Further studies should assess such interactions in the general population.

## Introduction

Psychiatric conditions manifest via signs and symptoms (American Psychiatric Association, 2000, 2013) that are closely connected to alterations of functioning (Galderisi *et al*., 2018), quality of life (QoL) (Hofer *et al*., 2017), medical comorbidities (Vancampfort *et al*., 2015, 2016; Stubbs *et al*., 2016*b*, 2017*a*; Correll *et al*., 2017) and life-style/behavioural habits, including nutrition, physical activity and substance abuse (Schuch *et al*., 2016; Stubbs *et al*., 2016*a*, 2018*a*, 2018*b*; Shivappa *et al*., 2018).

Several lines of evidence have implicated significant associations between functioning and QoL on the one hand and demographic, social, environmental, medical and psychiatric variables. For example, depressive symptoms have been associated with impaired functioning (Licht-Strunk *et al*., 2009), particularly in older populations (Ellervik *et al*., 2014), and with lower QoL (Lin *et al*., 2014). Also QoL appears to be associated with depression, ranging on a continuum from individuals without depression with higher QoL and functioning to patients with severe depression and lower QoL and functioning (Cotrena *et al*., 2016).

Additionally, a link has also been suggested between depression and multiple medical conditions. Several putative pathways have been proposed underlying this connection. For example, a bidirectional link has been described with obesity, with the two conditions possibly having a shared underlying biological pathway (Milaneschi *et al*., 2018; Vittengl, 2018). Among others, inflammatory cytokines and gut-hyperpermeability may be involved (Slyepchenko *et al*., 2016; Milaneschi *et al*., 2018). A higher prevalence of diabetes has been observed compared with the general population in patients with severe mental illness (SMI) (R1.85, 95% CI 1.45–2.37) (Vancampfort *et al*., 2016). The same is true for the metabolic syndrome (RR  =  1.58; 95% CI 1.35–1.86) (Vancampfort *et al*., 2015), as well as ultimately cardiovascular disease prevalence (O1.53, 95% CI 1.27–1.83) and incidence (H1.78, 95% CI 1.60–1.98) (Correll *et al*., 2017). Such high comorbidity figures often complicate medication prescriptions, ultimately resulting in polypharmacotherapy, with related drug–drug interactions and safety or tolerability issues (Ban *et al*., 1984; Frye *et al*., 2000).

In addition, life-style/behavioural habits play a relevant role in the onset and maintenance of both depression and medical comorbidities. Poor nutrition, among others, has been associated with depressive symptoms. While adherence to the Mediterranean diet is associated with better QoL (Veronese *et al*., 2016*a*), food habits with a higher dietary inflammatory index (DII^®^) are associated with an increased risk of depression (Shivappa *et al*., 2018). In addition to the already complex set of putative factors associated with depression, the educational level has also been associated with depression, or suicide attempts (Rahman *et al*., 2018). Moreover, substance use or abuse have also been associated with depression. For example, a robust association was observed between smoking and depression both in cross-sectional and prospective studies (Luger *et al*., 2014; Tjora *et al*., 2014; Stubbs *et al*., 2018*b*). Additionally, smoking was associated with increased stress levels(Stubbs *et al*., 2017*c*). However, despite the well-replicated direct association between smoking and depression and a negative association between depression and QoL, it has been reported that patients with SMI who smoke appear to have better mental QoL (Li *et al*., 2017). Such a paradox represents the complexity of the relationships among psychiatric symptoms, QoL and smoking, among other variables. Moreover, beyond smoking, an association between alcohol and depressive symptoms has also been described consistently (Choi *et al*., 2018; Hogarth *et al*., 2018).

Aforementioned associations are clinically relevant in particular in the population with or at risk of osteoarthritis, given the association between osteoarthritis and pain, osteoarthritis and depression(Veronese *et al*., 2017*c*), pain and depression (Stubbs *et al*., 2017*b*), in addition to the association between osteoarthritis and several other variables (Veronese *et al*., 2016*b*; Shivappa *et al*., 2018). Osteoarthritis associates with high social and health costs (Chen *et al*., 2012), and has led the World Health Organization to proclaim the past decade as the *Bone and Joint Decade* (2000–2010) (Woolf and Pfleger, 2003). Among comorbid conditions, depression brings huge costs (Chiu *et al*., 2017; Hsieh and Qin, 2018) also. Part of the costs of treatment of chronic conditions such as osteoarthritis and depression is also on patients’ shoulders, with a crucial role of income in determining treatment, outcome (Kemp *et al*., 2013) and ultimately additional costs related to poor outcome of osteoarthritis and depression. Research and prevention has been proposed as targets to tackle osteoarthritis-related burden (Chen *et al*., 2012). Indeed, understanding the relationship among factors that share common and reciprocal associations, such as depression, sedentary behaviour, smoking and alcohol consumption and other life-style parameters could allow to better understand where most efforts in prevention of several frequently comorbid disorders, or promotion of healthy life-style campaigns may be directed.

Given the aforementioned complex associations between depressive symptoms and other multidimensional factors, alternative statistical methods, such as network analysis, may better describe the reciprocal relationships among several variables, compared with standard statistical procedures. A network meta-analysis allows the simultaneous analysis of all relationships that may be important to a network of connected phenomena. Therefore, this approach has the advantage of providing a big picture of how several variables directly or indirectly interact in the whole person's clinical and non-clinical characteristics as well as absence of any *a priori* hypothesis, different from structural equation modelling. Network analysis models individual variables as nodes within a network, and correlations among variables that connect each node. For example, network analysis has been used to describe the reciprocal connections among positive and negative symptoms in schizophrenia, and functioning (Galderisi *et al*., 2018). Also, network analysis can individuate ‘bridging’ nodes that may connect other nodes within a network.

Based on the above, we aimed employing a network analysis to describe the complex interaction between age, body mass index (BMI), medical conditions, polypharmacotherapy, smoking and alcohol use, education, nutrition, depressive symptoms, functioning and QoL. We hypothesised that among all examined variables, depressive symptoms will play a central role in the studied networks. We further hypothesised that an aggregated measure of proneness to medical comorbidity will capture the relationship between physical and mental health.

## Methods

### Data source and subjects

Data were extracted from the freely available Osteoarthritis Initiative (OAI) database (http://www.oai.ucsf.edu/).

Within the OAI, potential participants were recruited across four clinical sites in the USA (Baltimore, MD; Pittsburgh, PA; Pawtucket, RI; and Columbus, OH) between February 2004 and May 2006. Inclusion and exclusion criteria of OAI are reported elsewhere (Eby and Eby, 2006; Shivappa *et al*., 2018). In brief, patients owith or at risk of knee osteoarthritis (but without end-state bilateral knee osteoarthritis), older than 45 years, with no ethnicity restriction, without inflammatory arthritis were included.

### Variable definitions

The variables included in the network were defined as follows:

Age (years) and BMI (kg/m^2^) (both continuous).

*Charlson comorbidity index (continuous).* Validated general health measures of self-reported comorbidities were assessed through a modified Charlson comorbidity score, with higher scores indicating an increased severity of conditions (Katz *et al*., 1996). Individual medical conditions used to build the Charlson comorbidity index were cerebrovascular disease, chronic obstructive pulmonary disease, gastro-intestinal ulcers, diabetes mellitus, asthma, heart failure, heart attack or to have undergone either percutaneous transluminal coronary angioplasty or coronary artery bypass, to have had bone fractures, knee osteoarthritis or cancer.

*Number of medications (continuous).* Number of medications was calculated based on the self-reported ongoing pharmacologic treatment.

*Alcohol (continuous).* Alcohol use was measured with the self-reported average number of drinks per week.

*Education (binary)*. Education was categorised as having completed college, or not.

*Smoking (binary).* Smoking was categorised as having ever smoked, or not.

*Income (binary)*. Income was categorised as having an income >US$50 000 per year, or not/not declared. Income was considered as a proxy of occupational functioning.

*Depressive symptoms (continuous).* Depressive symptoms were measured with the 20-item Center for Epidemiologic Studies-Depression (CES-D) self-rated instrument (Radloff, 1977). The range of possible values for this scores is 0–60, where higher scores indicate more depressive symptoms. We did not consider full-criteria depressive disorder or episode.

*Mediterranean diet adherence (continuous).* Adherence to Mediterranean diet was measured with the score proposed by Panagiotakos *et al.* (Panagiotakos *et al*., 2006; Veronese *et al*., 2016*a*, 2016*b*, 2017*a*).

*Physical activity (continuous)*. Physical activity was evaluated using the validated Physical Activity Scale for the Elderly (PASE). This scale covers 12 physical activities, scoring from 0 without a cut-off score.

*Health-related QoL (continuous)*. Health-related QoL was measured with the Short-Form Health Survey 12 (SF-12) (McHorney *et al*., 1994; Burdine *et al*., 2000), and in particular two separate scores were considered in the network: physical SF-12, namely the QoL related to physical health, and mental SF-12, namely the QoL related to mental health.

### Network estimation

All codes used for the present analyses are described in the online Supplementary Material.

The network was estimated with RStudio (R_Core_Team, 2013) using mgm and qgraph package according to the methods described by Borsboom and Cramer (2013), Costantini *et al*. (2014), Haslbeck and Waldorp (2015) and Epskamp *et al*. (2017).

Within the networks (Cuthbert, 2014; Insel, 2014; Wildes and Marcus, 2015; Patrick and Hajcak, 2016; Epskamp *et al*., 2017), variables are represented as nodes, connected by edges. Namely, edges are the regularised partial correlations between the nodes. Nodes are reciprocally connected creating a network of interacting self-reinforcing pools of variables. In the present network analysis, a pairwise mixed graphical model was estimated (Haslbeck and Waldorp, 2015) and several properties of the estimated network were measured (Epskamp *et al*., 2012). This method allows to estimate undirected associations, called edges, among variables, called nodes, without implying any directionality, and allowing to pool together categorical and continuous variables. Biological variables and other conditions are often interconnected, and an excess of sparse correlations may add confusion without adding information to a network interpretation. As a consequence, we applied a penalty to correlations close to zero, to retain only meaningful associations. Such operation is also defined as a ‘least absolute shrinkage and selection operator’ (LASSO) (Friedman *et al*., 2014) regularisation (a sort of shrinkage of small edges to zero), which was applied in order to only retain more solid *edges* (regularised partial correlations). Using LASSO, we aimed for a conservative approach that makes results more interpretable. Also, the Extended Bayesian Information Criterion (EBIC) (Chen and Chen, 2008; Foygel and Drton, 2010), a parameter that sets the degree of regularisation/penalty applied to sparse correlations, was set to 0.5 (BIC  =  0 would allow sparse and meaningless correlations to survive regularisation, while BIC  =  0.5 applies a conservative approach).

### Network inference

Analyses also assessed the centrality indices of nodes, namely how strongly the nodes were interconnected with several other nodes of the network. Centrality indices (node strength, closeness, betweenness) measure how important a node is in a given network. Centrality of nodes was estimated with node strength (i.e. the absolute sum of edge weights), closeness (i.e. the inverse of the sum of the distances of the focal node from all the other nodes in the network) (Costantini *et al*., 2014) and betweenness indices (i.e. the number of shortest paths between any two nodes that pass through the node of interest) (Costantini *et al*., 2014). This approach allows circularity of regularised partial correlations. Such methodology is considered an alternative approach to generalised linear modelling, or latent component analysis. No *a priori* direction or causal modelling is assumed, also because data used in the model were observational and cross-sectional.

### Network stability

The stability of the network after removing increasing percentages of patients was measured as well, by means of a case-dropping subset bootstrapping, namely the re-calculation of centrality indices after dropping growing percentages of the included participants (Epskamp *et al*., 2017). To quantify stability of the centrality indices, the correlation stability coefficient (CS) was calculated. CS represents the maximum proportion of population that can be dropped with re-calculated indices correlating at least 0.7 with indices of the original full sample (auto-correlation). Networks with reliable centrality should have a CS ≥0.25, ideally higher than 0.5 for centrality estimates, being optimal if 0.75. In addition, to measure edges’ accuracy, an estimated 95% confidence interval of the range containing the true regularised partial correlations (edge) was calculated by means of ‘non-parametric’ bootstrapping (*n* boots  =  1000).

## Results

### Characteristics of the included sample

Characteristics of the included sample are reported in Table 1. Out of 4796 individuals potentially eligible, we ultimately included 3532 North-American adults with or at risk for osteoarthritis. Excluded were subjects with missing values of at least one of the variables included in the network. Mean age of participants was 62.2 (9.0) years, and the average number of medications was 3.68 (2.55). Cases included in the present analysis had significantly higher Charlson comorbidity score (*p* < 0.001), more depressive symptoms (*p*  =  0.043), were more frequently smokers (*p* < 0.001), lower physical and mental health-related QoL (*p*  =  0.007; *p*  =  0.047, respectively), lower physical activity levels (*p*  =  0.013), lower income (*p* < 0.001) and slightly higher adherence to Mediterranean diet (*p* < 0.001), compared with those excluded for missing values. No difference emerged with respect to age, education, BMI, alcohol intake, number of medications.

**Table 1.** Characteristics of multidimensional network in a sample of 3532 North-American adults

Continuous variables	Mean	s.d.	Continuous variables	Mean	s.d.	Categorical variable	%
Age (years)	62.15	9.01	SF-12 physical (range: 13–68)	48.20	9.16	Education (college graduate)	30.18
Body mass index (kg/m^2^)	28.91	4.84	SF-12 mental (range: 10–73)	53.56	8.22	Lifetime smoking (yes)	48.50
CES-D (range: 0–57)	6.82	7.07	PASE (range: 0–531)	155.08	80.71	Income >US$50 000/year	60.11
Alcohol (number of drinks/week)	1.71	1.48	Number of medications	3.68	2.54		
Charlson index (range: 0–10)	0.45	0.89	Mediterranean diet adherence score (range: 0–44)	28.09	5.01		

CES-D, Center for Epidemiologic Studies-Depression Scale; COPD, chronic obstructive pulmonary disease; Education, completed college; Functioning, income >US$50 000; PASE, Physical activity Scale for the Elderly; SF-12, Short-Form Health Survey 12 items

### Network description

**Fig. 1.**
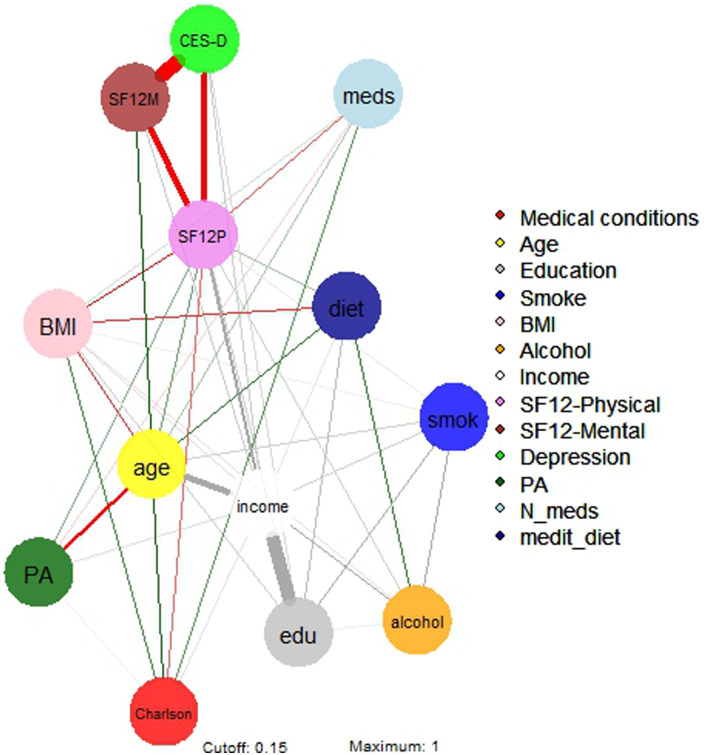
Network 1 of multidimensional variables in a sample of 3532 North-American adults aged >45 years old. BMI, body mass index; Depression, Center for Epidemiologic Studies – Depression score; drinkweek, drinks per week; education, college completers; Income, yearly income >US$50 000; Medical conditions, Charlson comorbidity index; *N*_meds, number of medications; medit_diet, adherence to Mediterranean diet; Physical activity, PASE – Physical activity Scale for the Elderly; SF12 phys/ment, Short-Form Health Survey 12 physical/mental score; Smoking, life-time smokers.

The regularised partial correlations matrix is reported in online Supplementary Table S1, and the network is represented in Figure 1.

#### Income

The strongest regularised partial correlations were observed between income and education (0.652), alcohol (0.186) and physical health-related QoL (0.232). Income also correlated with physical activity (0.056) and adherence to Mediterranean diet (0.041). Negative connections were found between income and age (−0.356), depressive symptoms (−0.091), Charlson comorbidity score (−0.049) and BMI (−0.056).

#### Physical health-related QoL

The strongest direct regularised partial correlations were observed between physical health-related QoL and income (0.232), followed by education and age (0.087), physical activity (0.073), Mediterranean diet (0.054) and alcohol (0.034). Negative connections were found between physical health-related QoL and depressive symptoms (−0.354), BMI (−0.146), number of medications (−0.104) and Charlson comorbidity index (−0.095).

#### Age

The strongest regularised partial correlations were observed between age and mental health-related QoL (0.173), Charlson comorbidity index (0.146), adherence to Mediterranean diet (0.126), smoking (0.081) and polypharmacotherapy (0.052). Negative connections were found between age and income (−0.355), physical activity (−0.263) and BMI (−0.129).

#### Mental health-related QoL

The strongest regularised partial correlations were between mental health-related QoL and age (0.173), and income (0.090). Negative connections were found between mental health-related QoL and depressive symptoms (−0.702).

#### Depressive symptoms

Negative connections were found between depressive symptoms and mental health-related QoL (−0.702), physical health-related QoL (−0.354), income (−0.090) and education (−0.068).

#### Education

The strongest regularised partial correlations were observed between education and income (0.652), smoking (0.142) and adherence to Mediterranean diet (0.116). A negative connection was found between education and depressive symptoms (−0.068).

To be noted, physical health-related QoL was a ‘bridge node’ among depressive symptoms and several other nodes, including Charlson comorbidity index, polypharmacotherapy, BMI, physical activity and adherence to Mediterranean diet.

### Network inference

**Fig. 2.**
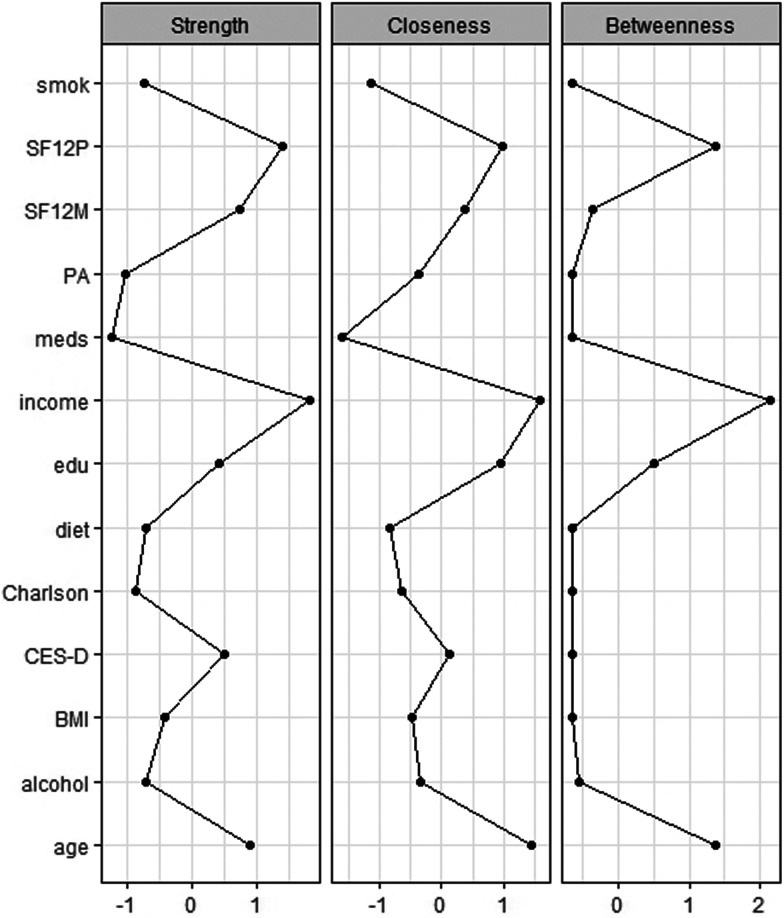
Centrality indices of multidimensional variables in a sample of 3532 North-American adults aged >45 years old. BMI, body mass index; Ch_, Charlson comorbiditiy index; cll, college completers; CES, Center for Epidemiologic Studies-Depression; drn, drinks per week; *i* > 5, yearly income >US$50 000; mds, number of medications; md_, adherence to Mediterranean diet; PAS, PASE – Physical activity Scale for the Elderly; SF12p/SF12m, Short-Form Health Survey 12 physical/mental score, smk, life-time smokers.

The network's representation is summarised in Fig. 2. Strengths of the multidimensional nodes are reported in Table 2, and figures of centrality indices are reported in Fig. 2. Among nodes with higher centrality indices (higher than *M*  =  1), the most central node was income (*M*  =  1.891), followed by physical health-related QoL (*M*  =  1.681), age (*M*  =  1.416), mental health-related QoL (*M*  =  1.342), depressive symptoms (*M*  =  1.214) and education (*M*  =  1.173). Lower centrality indices between 0.5 and 0.8 were found for BMI (*M*  =  0.733), alcohol use (*M*  =  0.590), adherence to Mediterranean diet (*M*  =  0.587), smoking (*M*  =  0.571) and Charlson comorbidity index (*M*  =  0.512).

**Table 2.** Strength of nodes of multidimensional networks in a sample of 3532 North-American adults years old

Nodes	Strength
Income >US$50 000	1.891
Short-Form Health Survey 12 items (SF-12) physical health score	1.681
Age	1.416
Short-Form Health Survey 12 items (SF-12) mental health score	1.342
Center for Epidemiologic Studies-Depression (CES-D) score	1.214
College graduate	1.173
Body mass index	0.733
Mean alcoholic drinks per week	0.590
Mediterranean diet adherence score	0.587
Lifetime smoking status	0.571
Charlson comorbidity index	0.512
Physical activity Scale for the Elderly (PASE) score	0.431
Number of medications	0.311

### Network stability

**Fig. 3.**
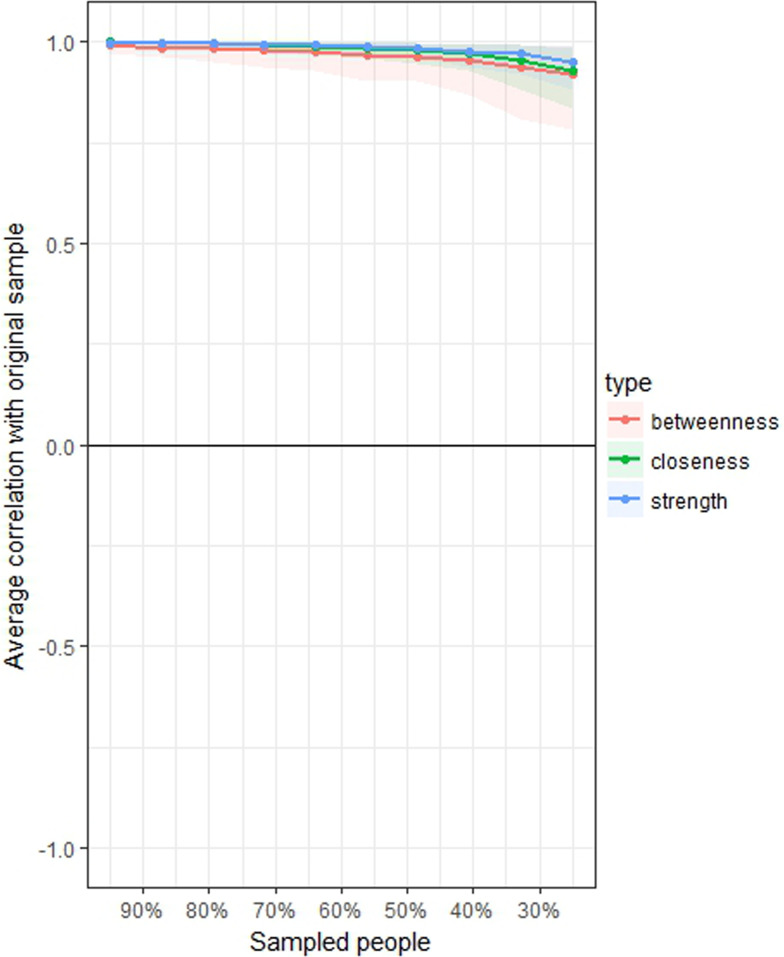
Average correlations between centrality indices of networks sampled with persons dropped and in the original sample of 3532 North-American adults aged >45 years old. Lines indicate the means and areas indicate the range from the 2.5th quantile to the 97.5th quantile.

Stability of the network 2, measured with the central stability coefficient (maximum drop proportions to retain correlation of 0.7 in at least 95% of the sample) was 0.75 or above for strength, 0.75 or above for closeness and 0.75 or above for betweenness (Fig. 3). Accuracy of the estimated edges among nodes is represented in online Supplementary Fig. S1.

## Discussion

The aim of this study was to describe the complex interactions of a multidimensional set of variables in a population of North-American adults with or at risk for knee osteoarthritis. A further aim was to test whether depressive symptoms had a central role in this network, and whether depressive symptoms had a regularised partial correlation with life-style and health variables. Importantly, all associations reported in results and discussed below in this section have no direction, and preclude any causal inference.

Physical and mental health-related QoL, age, income, education and depressive symptoms were the variables with higher centrality. Nodes with higher centrality are those that maintain the status of the network, and those whose modification is expected to influence the whole network (Costantini *et al*., 2014).

Physical health-related QoL was associated with both physical activity and Mediterranean diet, as well as with income and education. These associations may support the central role of policies investing in education to reduce health-related costs in later stages of life (AHRQ, 2015; Hahn and Truman, 2015). Furthermore, this result confirms previous findings supporting the role of physical activity in improving QoL (Rejeski and Mihalko, 2001; Penedo and Dahn, 2005), and the role of exercise in improving QoL even in people affected by depression (Schuch *et al*., 2016). Moreover, the positive association between Mediterranean diet and QoL is in line with results from several large cohort studies (Henriquez Sanchez *et al*., 2012; Costarelli *et al*., 2013; Milte *et al*., 2015; Veronese *et al*., 2016*a*). The association between income and higher physical health-related QoL is also in line with previous evidence from clinical and general populations (Costa and Nogueira, 2014; Wyshak, 2016).

Mental health-related QoL was higher in older patients, again being consistent with previous findings in the general population (Bell, 2014). Reduced life expectancy in people with SMI (Chang *et al*., 2011; Laursen *et al*., 2013; Correll *et al*., 2017) might have also played a role in decreasing the rates of subjects with mental illness among elderly subjects. Additionally, the direct association between income and mental health-related QoL is also consistent with previous findings(Cao *et al*., 2016).

Income in the analysed sample was associated with a large set of favourable health and life-style outcomes, namely education, QoL, physical activity and adherence to Mediterranean diet. Replication of this association may differ across countries other than USA, given the different welfare organisations that may influence general well-being, access to health-care and QoL. However, to the best of our knowledge, we are not aware of any other study applying the same analysis to samples from different countries. Also, the direction of such associations remains to be elucidated.

As hypothesised, depressive symptoms played a central role in the analysed networks. In particular, CES-D score was indirectly correlated with Charlson comorbidity index, polypharmacotherapy and BMI. These three regularised partial correlations strongly support the proneness of individuals with depressive symptoms to be affected by medical illnesses or *vice versa*, and in particular suggest that mental health-related QoL may play a role in bridging mental and medical illness. These results are consistent with the large body of research supporting an increased medical burden in patients with SMI (Vancampfort *et al*., 2015; Vancampfort *et al*., 2016; Correll *et al*., 2017; Stubbs *et al*., 2017*a*), possibly extending it to sub-clinical populations. According to this network analysis, the association (yet indirect) between physical illness and depressive symptoms holds true in the elderly OAI population as well, in addition to clinical populations with full-criteria diagnosed depressive disorder. In other words, it appears that the association between depressive symptoms and medical comorbidity may already exist in the presence of sub-clinical depressive symptoms, and that it may be mediated by QoL.

Furthermore, this network analysis observed an indirect association between depressive symptoms and a general proneness to medical comorbidity. Several reasons may explain why a cumulative index of medical comorbidities was indirectly associated with depressive symptoms. First, common pathways have been described among depression and medical conditions, such as a pro-inflammatory state (Slyepchenko *et al*., 2016; Kohler *et al*., 2017; Milaneschi *et al*., 2018). Also, unhealthy life-style behaviours are common/non-specific risk factors for both depression and several medical conditions (Stubbs *et al*., 2016*a*; Veronese *et al*., 2016*a*, 2017*b*, 2017*d*; Soysal *et al*., 2017; Shivappa *et al*., 2018). Clinical implications of the close connection between depressive symptoms and greater morbidity and mortality from medical illnesses may exist (Vancampfort *et al*., 2015, 2016; Correll *et al*., 2017; Stubbs *et al*., 2017*a*).

These findings are relevant for several reasons. First, screening and early interventions for both depressive symptoms and medical conditions should be considered in health-care services (Mitchell *et al*., 2014; Mitchell *et al*., 2015). Second, should mental illness occur, it has been proposed that higher levels of physical comorbidity complicate the successful treatment of patients with depression (Sato and Yeh, 2013), given the additional safety and tolerability issues of psychopharmacologic agents in case of comorbid diabetes, cardiovascular disease, arthritis or chronic obstructive pulmonary disease (Stubbs *et al*., 2016*b*; Solmi *et al*., 2017). Third, in case of medical comorbidity, polypharmacotherapy is the rule rather than an exception, with drug–drug interactions further complicating the efficacy and safety scenario. Also, a finer grained description of the complex interaction among variables included in the network could help in better understanding what ongoing associations are there when designing specific interventions. This could be relevant in particular in the case of patients with osteoarthritis. People with osteoarthritis often suffer from chronic pain (Ho-Pham *et al*., 2014), which limits physical activity and increases sedentary behaviour. Low physical activity is associated with weight gain, which in turn is associated with a large set of unfavourable health outcomes. Also, depression itself is associated with low levels of physical activity and multimorbidity (Stubbs *et al*., 2017*a*). Hence, understanding the complex interaction among such variables, and showing that they are interconnected in a stable network, may suggest to design future interventions which ideally should not scotomise such a complex scenario. Moreover, the bridging role of physical health-related QoL may be targeted by specific interventions to potentially weaken the apparently indirect association among depressive symptoms and medical comorbidities, polypharmacotherapy, BMI, physical activity and adherence to Mediterranean diet. Of course, studies with a longitudinal, ideally prospective design are needed to better characterise the direction of aforementioned associations, which remain unknown based on this network analysis.

The study has several strengths. First the network proved to be stable as measured by CS > 0.25; actually results yielded the highest possible stability coefficient (CS  =  0.75 for all centrality indices). This finding supports the reliability of the estimates of the multidimensional network among the variables included in the analysis, and should encourage the application of network analysis to other multidimensional sets of variables in future projects. Network analysis provides a valuable tool to investigate and describe how life-style, social, environmental, medical and psychiatric variables are closely connected. Second, the study had a large sample. Third, the network provides insight into a multidimensional set of variables in North-American elderly adults with or at risk for knee osteoarthritis who were not selected for mental health issues, providing insights into the connection between nutrition, social/occupational status functioning, depressive symptoms and other clinical and non-clinical variables.

The study has also some limitations that need to be considered when interpreting the results. First, several variables included in the network were self-reported (individual comorbidities that are summarised by the Charlson comorbidity index). Second, only depressive symptoms were measured, limiting any further consideration on other mental health burden, such as anxiety, bipolar disorder or psychosis spectrum symptoms. Third, depressive symptoms were measured with a rating scale, but no full-criteria diagnosis of depressive disorder was available. Fourth, data were cross-sectional, and therefore no causal inference can be made. Fifth, OAI includes adults aged >45 years old with knee osteoarthritis or at high risk of this condition. Finally, by excluding subjects with missing data, we selected a population with unfavourable medical and mental health status, which furtherly precludes any inference of present results to general population. Even if knee OA and its risk factors are very common also in the general population, other studies are needed to apply our results to the general population or different subpopulations of interest.

In conclusion, in elderly North-American adults with or at risk for osteoarthritis depressive symptoms play a central role in a multidimensional network, including QoL, income, age and medical comorbidities. The indirect association between the Charlson comorbidity index and polypharmacotherapy with depressive symptoms which are connected by a bridging node, namely health-related QoL, support the close interplay between these important variables that are each amenable to therapeutic action. QoL, income and age also play a central role. Finally, physical activity and adherence to Mediterranean diet were significantly associated with physical health-related QoL and with income. Future studies may address whether the same associations hold true in general population.

## Supplementary material

For supplementary material accompanying this paper visit http://dx.doi.org/10.1017/S204579601800077X. click here to view supplementary material

## Availability of data and materials

The original data set for the OAI is available from the OAI website (https://oai.epi-ucsf.org/datarelease/).
