# Network analysis of the relationship between depressive symptoms, demographics, nutrition, quality of life and medical condition factors in the Osteoarthritis Initiative database cohort of elderly North-American adults with or at risk for osteoarthritis – CORRIGENDUM

**DOI:** 10.1017/S2045796019000064

**Published:** 2019-03-08

**Authors:** Marco Solmi, Ai Koyanagi, Trevor Thompson, Michele Fornaro, Christoph U Correll, Nicola Veronese

**Affiliations:** 1 Neuroscience Department, Psychiatry Unit, University of Padua, Padua, Italy; 2 Padua University Hospital, Padua, Italy; 3 Neuroscience Center, University of Padua, Padua, Italy; 4 Instituto de Salud Carlos III, Centro de Investigación Biomédica en Red de Salud Mental, CIBERSAM, Monforte de Lemos 3-5 Pabellón 11, Madrid 28029, Spain; 5 Research and Development Unit, Parc Sanitari Sant Joan de Déu, Universitat de Barcelona, Fundació Sant Joan de Déu, Dr Antoni Pujadas, 42, Sant Boi de Llobregat, Barcelona 0883, Spain; 6 Faculty of Education and Health, University of Greenwich, London, UK; 7 Neuroscience Department, Section of Psychiatry, Federico II University of Naples, Naples, Italy; 8 Department of Psychiatry, The Zucker Hillside Hospital, Northwell Health, Glen Oaks, NY, USA; 9 Department of Psychiatry and Molecular Medicine, Hofstra Northwell School of Medicine, Hempstead, NY, USA; 10 Department of Child and Adolescent Psychiatry, Charité Universitätsmedizin, Berlin, Germany; 11 Ageing Branch, National Research Council, Padua, Italy

The above article was originally submitted to Epidemiology and Psychiatric Sciences with the incorrect author name listed for the second author. The correct surname for the second author is Ai Koyanagi (and not Ai Konayagi). The authors would like to apologise for this error.
