# Supplementary material for: Network analysis of the relationship between depressive symptoms, demographics, nutrition, quality of life and medical condition factors in the Osteoarthritis Initiative database cohort of elderly North-American adults with or at risk for osteoarthritis
Source: Epidemiol Psychiatr Sci. 2019 Feb 6;29:e14. doi: 10.1017/S204579601800077X (PMC8061212; doi:10.1017/S204579601800077X)
Supplement: Supplementary file 1 [file S204579601800077Xsup001.docx]

**Supplementary material**

Supplementary Figure 1. Bootstrapped confidence intervals of estimated edge-weights for the estimated network of multidimensional variables in a sample of 3,532 North-American adults aged > 45 years old.


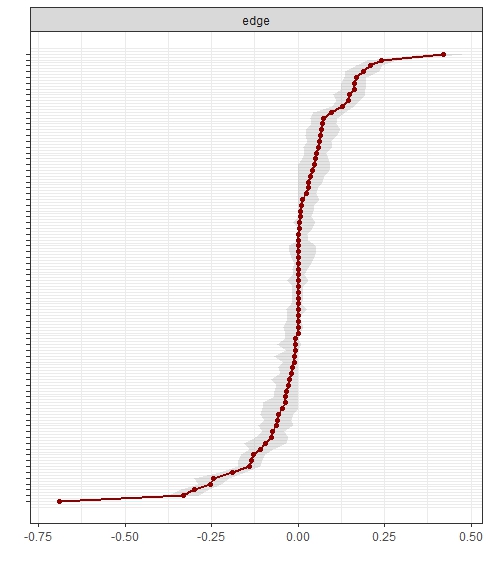


Supplementary Table 1. Regularized partial correlations matrix of multidimensional variables in a sample of North-American elderly adults from the Osteoarthritisi Initiative.

|  | Chalrson | age | college | smoking | bmi | drinkweek | income>51k | SF12phys | SF12ment | CESD | PASE | meds | med_diet |
| --- | --- | --- | --- | --- | --- | --- | --- | --- | --- | --- | --- | --- | --- |
| Chalrson | 1 |  |  |  |  |  |  |  |  |  |  |  |  |
| age | 0,146561 | 1 |  |  |  |  |  |  |  |  |  |  |  |
| college | 0 | 0 | 1 |  |  |  |  |  |  |  |  |  |  |
| smoking | 0 | 0,081343 | 0,142469 | 1 |  |  |  |  |  |  |  |  |  |
| bmi | 0,113269 | -0,12964 | -0,08353 | 0,025417 | 1 |  |  |  |  |  |  |  |  |
| drinweek | 0 | 0 | 0,023452 | 0,203734 | -0,02186 | 1 |  |  |  |  |  |  |  |
| income | -0,04925 | -0,35577 | 0,652434 | 0,082325 | -0,05499 | 0,18585 | 1 |  |  |  |  |  |  |
| SF12 Physical | -0,09495 | 0,087338 | 0,087177 | -0,03615 | -0,14652 | 0,034327 | 0,232607 | 1 |  |  |  |  |  |
| SF12 Mental | 0 | 0,173416 | 0 | 0 | 0 | 0 | 0,090164 | -0,37639 | 1 |  |  |  |  |
| CES-D | 0 | 0 | -0,06792 | 0 | 0 | 0 | -0,09057 | -0,3543 | -0,70218 | 1 |  |  |  |
| PASE | -0,0099 | -0,26365 | 0 | 0 | 0 | 0 | 0,056566 | 0,073669 | 0 | 0 | 1 |  |  |
| meds | 0,098636 | 0,051932 | 0 | 0 | 0,029495 | 0 | 0 | -0,10408 | 0 | 0 | -0,02751 | 1 |  |
| med_diet | 0 | 0,126733 | 0,116166 | 0 | -0,12835 | 0,12166 | 0,041025 | 0,053762 | 0 | 0 | 0 | 0 | 1 |

Legend. Charlson, Charlson comorbiditiy index; bmi, body mass index; college, college completers; CESD, Center for Epidemiologic Studies-Depression; drinkweek, alcohol drinks per week; income>50k, yearly income >50,000 U.S. dollars; meds, number of medications; med_diet, adherence to Mediterranean diet; PASE, Physical activity Scale for the Elderly; SF12 phys / ment, Short-Form Health Survey 12 physical / mental score; smoking, life-time smokers.

**Codes used for network analyses with RStudio Version 1.0.153.**

#set working directory

setwd("F:/Docs/Original_Papers/Network_OAI”)

#donwload and attach some packages to working environment

library(readxl)

install.packages("qgraph")

library("qgraph")

library("bootnet")

library("IsingFit")

library("IsingSampler")

library("graphicalVAR")

library("mlVAR")

library("reshape2")

library("ggplot2")

library("lavaan")

library("lme4")

library("glmnet")

library("depmixS4")

library("huge")

library("BayesFactor")

library("ltm")

library("ega")

library(mgm)

########################################

#OAI – network analysis

#Import file

all <- read_excel("F:/Docs/Original_Papers/Network_OAI/network2.xls")

#View file

View(all)

# remove missing data

allomit <- na.omit(all)

#View file without missing data

View(allomit)

#Estimate mixed graphical model

allomit_type <- c("p", "g", "c", "c", "g", "p", "c", "g", "g", "g", "g", "p", "g")

allomit_level <- c(1, 1, 2, 2, 1, 1, 2, 1, 1, 1, 1, 1, 1)

fitOAI <- mgm(data = allomit, type = allomit_type, level = allomit_level, scale = TRUE, k = 2, lambdaSel = "EBIC", lambdaGam = 0.25)

#export matrix

library(xlsx)

write.xlsx(fitOAI$pairwise$wadj, "cormat.xlsx")

#Assign names to variables

names(allomit) <- c ("Charlson", "age", "edu", "smok", "BMI", "alcohol", "income", "SF12P", "SF12M", "CES-D", "PA", "meds", "diet")

#Plot network and estimate properties

Graph_lassoall <- qgraph(fitOAI$pairwise$wadj, edge.color = fitOAI$pairwise$edgecolor, layout = "spring", tuning = 0.5, sampleSize = nrow(allomit), minimum = 0, maximum = 1, details = TRUE, esize = 20, lambda.min.ratio = 0.01, cut = 0.15, labels = names(allomit), labels.cex = 12, groups = list("Medical conditions" = 1:1, "Age" = 2:2, "Education" = 3:3, "Smoke" = 4:4,"BMI" = 5:5, "Alcohol" = 6:6, "Income" = 7:7,"SF12-Physical" = 8:8, "SF12-Mental" = 9:9, "Depression" = 10:10, "PA" = 11:11, "N_meds" = 12:12, "medit_diet" = 13:13), legend.cex = 0.45, vsize = 8, esize = 25, pastel = TRUE, posCol = "blue", negCol = "red",color = c("red", "yellow", "grey", "blue", "pink", "orange", "white", "violet", "brown", "green", "dark green", "light blue", "dark blue"), borders = FALSE, vTrans = 200, details = TRUE)

# Compute and plot centrality index

centResall <- centrality(Graph_lassoall)

centResall$OutDegree

centResall$Closeness

centResall$Betweenness

centralityPlot(Graph_lassoall)

#Edges 95% confidence intervals

resultsall <- estimateNetwork(allomit, default = "EBICglasso", corMethod = "cor_auto", tuning = 0.5)

boot1all <- bootnet(resultsall, nBoots = 2500,nCores = 8, type = "nonparametric")

plot(boot1all, labels = FALSE, order = "sample")

print(boot1all)

summary(boot1all)

# Network stability

networkall <- estimateNetwork(allomit, default = "EBICglasso", corMethod = "cor_auto", tuning = 0.5, refit = TRUE)

boot2all <- bootnet(networkall, nBoots = 2500,type = "case", nCores = 8)

plot(boot2all)

print(boot2all)

summary(boot2all)

# CS central stabilit coefficient (should be above 0.25, and is good if >0.5)

corStability(boot2all)
